# Effects on mitochondrial transcription of manipulating mTERF protein levels in cultured human HEK293 cells

**DOI:** 10.1186/1471-2199-11-72

**Published:** 2010-09-16

**Authors:** Anne K Hyvärinen, Mona K Kumanto, Sanna K Marjavaara, Howard T Jacobs

**Affiliations:** 1 Institute of Medical Technology and Tampere University Hospital, FI-33014 University of Tampere, Finland; 2 Research Program of Molecular Neurology, FI-00014 University of Helsinki, Finland

## Abstract

**Background:**

Based on its activities *in vitro*, the mammalian mitochondrial transcription termination factor mTERF has been proposed to regulate mitochondrial transcription by favouring termination at its high-affinity binding immediately downstream of the rDNA segment of mitochondrial DNA, and initiation selectively at the PH1 site of the heavy-strand promoter. This defines an rDNA transcription unit distinct from the 'global' heavy-strand transcription unit initiating at PH2. However, evidence that the relative activities of the two heavy-strand transcription units are modulated by mTERF *in vivo *is thus far lacking.

**Results:**

To test this hypothesis, we engineered human HEK293-derived cells for over-expression or knockdown of mTERF, and measured the steady-state levels of transcripts belonging to different transcription units, namely tRNA^Leu(UUR) ^and ND1 mRNA for the PH2 transcription unit, and tRNA^Phe ^plus 12S and 16S rRNA for the PH1 transcription unit. The relative levels of 16S rRNA and ND1 mRNA were the same under all conditions tested, although mTERF knockdown resulted in increased levels of transcripts of 12S rRNA. The amount of tRNA^Phe ^relative to tRNA^Leu(UUR) ^was unaffected by mTERF over-expression, altered only slightly by mTERF knockdown, and was unchanged during recovery from ethidium bromide-induced depletion of mitochondrial RNA. mTERF overexpression or knockdown produced a substantial shift (3-5-fold) in the relative abundance of antisense transcripts either side of its high-affinity binding site.

**Conclusions:**

mTERF protein levels materially affect the amount of readthrough transcription on the antisense strand of mtDNA, whilst the effects on sense-strand transcripts are complex, and suggest the influence of compensatory mechanisms.

## Background

**Figure 1:**
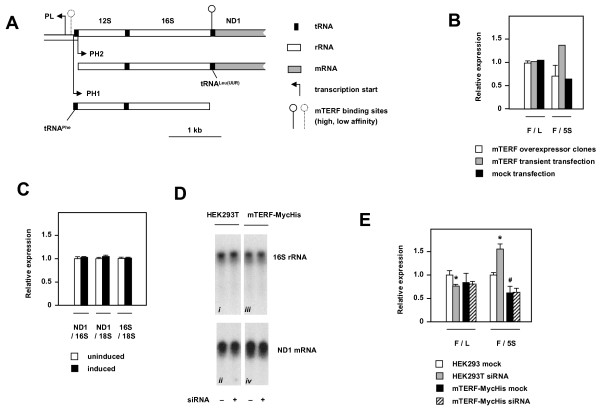
**Manipulation of mTERF expression has minimal effects on steady-state levels of mature mitochondrial RNAs**. (A) Schematic diagram of the promoter and rDNA region of human mtDNA. Because mTERF binding dictates the use of alternate transcriptional start sites and terminators, tRNA^Phe ^and tRNA^Leu(UUR) ^fall into separate transcription units (PH1 and PH2 respectively). (B) Relative expression of mitochondrial transcripts in cells overexpressing mTERF, based on phosphorimaging of Northern blots probed successively for mitochondrial tRNA^Phe ^and tRNA^Leu(UUR) ^and for 5S rRNA. Data (means ± SD) are signal ratios of tRNA^Phe ^to tRNA^Leu(UUR) ^(F/L) and tRNA^Phe ^to 5S rRNA (F/5S) for the mTERF-overexpressing clones shown in Additional File 1, Fig. S1, normalized to the corresponding ratio in cells stably transfected with empty-vector. Bars shown alongside are based on single reference experiments, using HEK293T cells transiently transfected with the same construct, or mock-transfected. (C) Q-RT-PCR analysis (means ± SD) of mitochondrial transcript levels, plus cytosolic18S rRNA, as indicated, in Flp-In™ T-Rex™-293 cells over-expressing mTERF-MycHis after doxycyclin induction for 3 d (or not induced). Data were normalized, in each case, to the corresponding ratio for uninduced cells. (D) Relative expression of mitochondrial transcripts in cells knocked down for mTERF, as indicated. – denotes mock-transfection. Northern blot probed successively for 16S rRNA and ND1 mRNA, as shown. The panels represent non-adjacent pairs of lanes from the same exposure of the same gel. (E) Relative expression of mitochondrial transcripts in cells knocked down for mTERF, as indicated, calculated from Northern blot data as in (B), normalized to the corresponding ratio in mock-transfected HEK293T cells. * indicates significant differences from the corresponding mock-transfected cells, and ^# ^a significant difference between cell-lines (*t*-test, *p *values as in text). For original blots see Additional File 1, Fig. S1C. Note that additional Q-RT-PCR data on levels of 12S rRNA gene transcripts are shown in Fig. 3.

Mammalian mitochondrial DNA is organized into three multicistronic transcription units (reviewed in [1], Fig. 1A), which give rise to the mature RNAs encoded by the circular genome: two ribosomal RNAs, 22 tRNAs and 11 mRNAs (2 of them bicistronic). Each strand is transcribed in its entirety, employing closely spaced promoters located within the major non-coding region of the genome, namely LSP, the promoter of the light-strand, with a unique initiation site designated PL, and PH1 and PH2, the alternate transcription start sites of the heavy-strand promoter (HSP), which give rise to partially overlapping transcripts. Based on metabolic labeling studies, PH1 and PH2 have been inferred to give rise to distinct primary transcripts of the heavy-strand [2]. PH1 is located within the non-coding region and generates a primary transcript comprising both rRNAs and two tRNAs (-Phe and -Val), terminating at the end of the rDNA region, mainly within the 5' end of the tRNA^Leu(UUR) ^gene [3]. PH2 is located within the coding sequence of tRNA^Phe ^and generates a primary transcript comprising all of the remaining heavy-strand encoded genes. PL generates a primary transcript comprising the entire light strand.

The mechanism by which the transcriptional machinery selects between these different initiation sites, and also effects selective termination at the end of the rDNA, in the case of transcripts initiated at PH1, is incompletely understood. It can be manipulated *in organello *by various drugs and by ATP [4-6]. The mitochondrial RNA polymerase comprises a single catalytic subunit, MTRPOL, plus an accessory factor, TFB2M, required for formation of the initiation complex *in vitro *at both HSP and LSP [7,8], together with mitochondrial transcription factor A (TFAM), which is needed for promoter-dependent transcription *in vitro *[7,9]. TFAM has a natural binding affinity for DNA and has been suggested also to play a more general role in organizing the mitochondrial chromosome, analogous with bacterial HU or eukaryotic and archaeal histones. A third factor, mTERF, with sequence-specific binding affinity for a sequence located within the tRNA^Leu(UUR) ^gene immediately downstream of the rDNA [10,11], has been proposed to play a key role in both initiation and termination of the PH1 transcription unit [12].

mTERF has selective termination activity *in vitro *on templates containing its high-affinity binding site in the tRNA^Leu(UUR) ^gene [10,13]. In crude extracts [14], as well as in a reconstituted system based on recombinant proteins [15], this activity appears to be bidirectional, but operates in the latter case more efficiently in the reverse direction, i.e. to terminate transcription initiating from the LSP side more efficiently than from HSP [15]. Based on the fact that it has weak binding to other sites in mtDNA, including the promoter region [12,16,17], it has been proposed that mTERF favours transcription of the PH1 transcription unit by simultaneous binding to the promoter and to the terminator region, creating a loop structure that can be visualized *in vitro *[12]. The level of active mTERF would thus act as a fine tuning of the relative production of rRNA and mRNA.

There are, however, some problems associated with this model. First, efficient transcription from PH1 *in vitro *does not require mTERF (although does appear to be stimulated by it [18]), whereas transcription from PH2 *in vitro *is weak [18]. Second, measurements of the relative half-lives of mitochondrial rRNAs and mRNAs in cultured cells [19] indicate that post-transcriptional regulation is substantial and may in fact be sufficient to maintain the different transcript levels seen *in vivo*, without the need for any differential regulation of transcription from the PH1 and PH2 transcription units. Note that, although the synthesis rates of mitochondrial rRNAs and mRNAs appear to be very different in both cultured cells [19] and rat liver [20], 'synthesis rate' here includes RNA processing as well as transcription. *In organello*, the combined rate of accumulation of pre-rRNA plus mature rRNA is, in fact, lower than that of mRNA [6]. Third, no modulation of transcription from the two initiation sites correlating with mTERF activity has ever been convincingly demonstrated *in vivo*. Fourth, in cells bearing the 3423A > G mutation, which greatly impairs mTERF binding *in vitro*, there is no alteration in the relative levels of 16S rRNA and ND1 mRNA [21,22], and no alteration in site occupancy *in vivo*, based on footprinting studies [21]. Fifth, decreased levels of mTERF expression in *Mpv17 *knockout mice are associated with globally increased mitochondrial transcription [23], suggesting rather than mTERF may function *in vivo *as a negative but general regulator of transcription. Finally, whilst recombinant mTERF is active in a reconstituted system *in vitro *[15], its activity in the presence of less pure mitochondrial extracts is subject to post-translational modifications and/or the presence of other proteins [11-13,18,24], raising doubts as to whether and how it influences transcription *in vivo*.

mTERF is a member of a family of organellar proteins proposed to interact with DNA to produce a variety of outcomes [25]. In mammals, two homologues of mTERF, MTERFD1 (mTERF3) and MTERD3 (mTERF2), have been shown to influence mitochondrial RNA levels and have been proposed to act as regulators of transcription from LSP [26,27], with consequent effects on oxidative phosphorylation mediated brought about by altered translation, as seen also in *Drosophila *[28]. However, neither mTERF homologue has been conclusively demonstrated to have high-affinity sequence-specific binding to DNA [26,27,29]. Homologues of mTERF in invertebrates have been demonstrated to influence both RNA and DNA synthesis *in vitro*, but here too, there is only weak evidence for a specific role *in vivo*. The mTERF-homologue in sea urchins, mtDBP, binds to at least two sites in the mitochondrial genome [30] and exhibits bidirectional transcription termination activity *in vitro *in the presence of human mitochondrial RNA polymerase, although it acts unidirectionally in combination with phage polymerases [31]. It also impedes the progress of DNA polymerase bidirectionally, acting as a contrahelicase *in vitro *[32], suggesting a possible role in DNA replication. A role for mTERF in mammalian mtDNA replication is also suggested by the observation that the level of mTERF expression in cultured human cells influences replication pausing in the vicinity of mTERF binding sites [16]. The *Drosophila *mTERF homologue mTTF binds to two putative transcriptional terminators [33], acting *in vitro *with similar directional properties to mtDBP [34]. Manipulation of DmTTF levels in cultured cells leads to effects on transcript levels consistent with it acting in the manner hypothesized for mTERF, i.e. as a regulator of termination (bidirectionally) and also of promoter activity [35].

The difficulty of interpreting *in vitro *experiments, and the open questions regarding the role of mTERF *in vivo*, prompted us to address the issue of whether and how mTERF activity influences mitochondrial RNA levels in cultured human cells. Clearly, if mTERF is a regulator of mitochondrial transcription *in vivo*, via a model as proposed, up- or down-regulation of its expression should influence mitochondrial RNA levels in a predictable fashion. We therefore undertook a study of mitochondrial transcripts in cells over-expressing or knocked down for mTERF. Surprisingly, we found that varying the level of mTERF over a wide range has only a small effect on the levels of sense-strand transcripts of the mitochondrial genome in the rDNA region. Conversely, we detected a clear effect on the relative amounts of antisense transcripts on the two sides of the high-affinity binding site. These findings support a role for mTERF in influencing mitochondrial transcription *in vivo*, but not in setting the levels of mature mitochondrial transcripts.

## Results

### Over-expression of mTERF does not alter steady-state levels of mature mitochondrial RNAs

To evaluate whether the expression level of mTERF influences the steady-state levels of the mature mitochondrial transcripts encoded on either side of its high-affinity binding site, we generated a series of transfected HEK293T cell clones stably over-expressing the natural mTERF protein. Expression of the mTERF transgene was verified at the RNA level by Q-RT-PCR (Additional File 1, Fig. S1A) and at the protein level by the substantial increase in DNA-binding capacity at the high-affinity mTERF binding site, as judged by EMSA (electrophoretic mobility shift assay, Additional File 1, Fig. S1B).

We analysed two parameters which we considered diagnostic for the relative utilization of the two heavy-strand transcription units predicted by the classic model of mammalian mtDNA transcription (Fig. 1A). The first is the relative amounts of tRNAs -Phe and -Leu(UUR), which are exclusively produced by transcription from PH1 and PH2, respectively, according to the classical model. The second is the relative amounts of mature 16S rRNA and ND1 mRNA. The latter is synthesized via transcription from PH2, whereas the former has been proposed to be generated mainly or exclusively from transcription initiating at PH1, although it has not been formally excluded that transcription from PH2 also contributes some of the 16S rRNA. We found that the relative amounts of tRNAs -Phe and -Leu(UUR) in different cell-clones over-expressing natural mTERF was indistinguishable from that in control cells transfected with empty vector (Fig. 1B), and was also unchanged in cells transiently transfected with the mTERF overexpression construct (or mock transfected cells). The global amount of mitochondrial transcription, as measured by the ratio of tRNA^Phe ^to cytosolic 5S rRNA was more variable, but showed no systematic relation to mTERF overexpression (Fig. 1B). We also found no detectable alteration in the relative amounts of mature NDI1 mRNA and 16S rRNA between mTERF over-expressing clones and control cells, based on Northern blots (Fig. 1D: compare lanes 1 of panels *i *and *ii *[control cells] with lanes 1 of panels *iii *and *iv *[over-expressing cells]). In an effort to quantify any such effect and avoid possible influences of cell background, we also used Q-RT-PCR to analyse transcripts of the 16S and ND1 genes in RNA extracted from Flp-In™ T-Rex™-293 cells stably transfected with the mTERF-MycHis construct, in which expression of mTERF can be induced by doxycycline (Fig. 1C). We found no differences in the relative amounts of transcripts from these two genes, nor in the ratio of either to cytosolic 18S rRNA transcripts.

### Effects of mTERF knockdown on steady-state levels of mature mitochondrial RNAs

In previous studies [16] we noted that transfection with an siRNA directed against mTERF suppressed most of the binding activity at the high-affinity mTERF binding site, as judged by EMSA [16]. We therefore compared the relative levels of mitochondrial transcripts in cells knocked down for mTERF. Northern blots probed successively for 16S rRNA and ND1 mRNA showed no difference in the relative levels of these mature transcripts in HEK293T cells after prolonged treatment (7 d) with an mTERF-specific siRNA (Fig. 1D: compare lanes 1 and 2 of panels *i *and *ii*), nor in mTERF-MycHis over-expressing cells knocked down for mTERF (Fig. 1D: compares lanes 1 and 2 of panels *iii *and *iv*). mTERF knockdown in HEK293T cells did, however, produce a small but significant decrease in the relative amount of tRNA^Phe ^compared with tRNA^Leu(UUR)^, (Fig. 1E, *t*-test, *p *< 0.05), accompanied by an increase in the overall amount of mitochondrial tRNAs, represented by the ratio of mitochondrial tRNA^Phe ^to cytosolic 5S rRNA (*t*-test, *p *< 0.01). siRNA treatment of mTERF-MycHis overexpressing cells caused no significant alteration in mitochondrial tRNAs (Fig. 1E), compared with mock-transfected cells. Note also that cells overexpressing mTERF-MycHis showed no clear difference from HEK293T cells in the relative levels of mitochondrial tRNA^Phe ^and tRNA^Leu(UUR) ^(Fig. 1E), although mitochondrial tRNA levels globally were lower than in untransfected HEK293T cells (*t*-test, *p *< 0.01).

### Manipulation of mTERF expression does not alter the relative levels of mitochondrial tRNAs during recovery from mitochondrial RNA depletion

**Figure 2:**
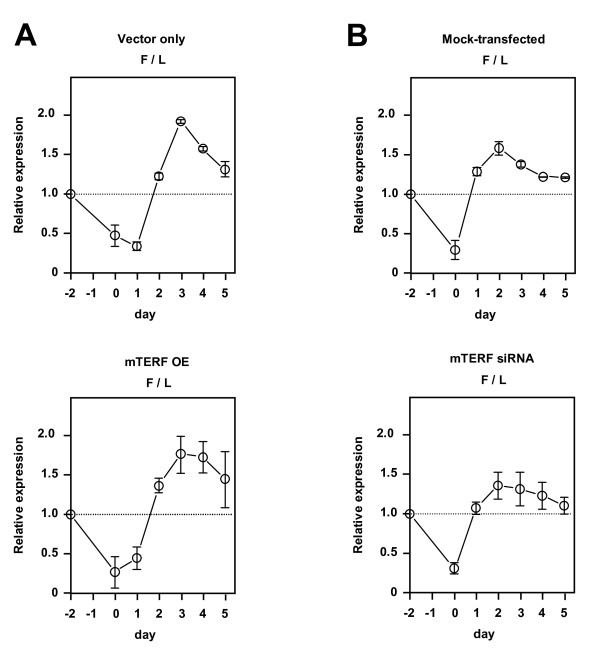
**Manipulation of mTERF expression has minimal effects on levels of mature mitochondrial RNAs during recovery from EtBr-induced depletion**. Relative expression of mitochondrial transcripts in cells overexpressing mTERF, based on phosphorimaging of Northern blots probed successively for mitochondrial tRNA^Phe ^and tRNA^Leu(UUR)^. Data (means ± SD) are ratios of tRNA^Phe ^to tRNA^Leu(UUR) ^(F/L) normalized to the ratio at the start of the experiment (time-point –2 d). (A) Cells stably transfected with empty-vector (as shown in Additional File 1, Fig. S1B) or mTERF overexpression (OE) construct (clone 3, as shown in Additional File 1, Fig. S1). Overexpressor clones 1 and 2 gave similar results: sample blots shown in Additional File 1, Fig. S2A. (B) Cells treated with mTERF-specific siRNA (or mock-transfected) prior to the addition of EtBr (day –2) and again 2 days after removal of EtBr (day 2). Days 1-5 indicate the period of subsequent recovery. For equivalent data on ratio of tRNA^Phe ^to 5S rRNA from the same experiment see Additional File 1, Fig. S2B, C.

Reasoning that the steady-state levels of mature mitochondrial transcripts may not accurately reflect their transcription rates *in vivo*, due to the influence of post-transcriptional processing, we set out to study whether the level of mTERF expression can influence the re-accumulation of tRNA transcripts belonging to the PH1 and PH2 transcription units during recovery from ethidium bromide (EtBr)-induced depletion of mitochondrial RNA. We compared the ratio of mitochondrial tRNAs -Phe and -Leu(UUR) in stably transfected cells overexpressing mTERF-with that in empty vector-transfected cells over 2 days of EtBr treatment followed by 5 days of recovery (Fig. 2A, Additional File 1, Fig. S2A). In both cell lines the ratio fell substantially during depletion, reflecting the much shorter half-life of tRNA^Phe^, but then recovered to levels higher than those seen in untreated cells, before decreasing again gradually, towards the starting value. This may indicate that the PH2 transcription unit is used preferentially during recovery from depletion. However, this did not appear to be influenced by the level of mTERF, since the same pattern was seen in control cells and in three separately analysed overexpressor cell lines, as well as in cells knocked down for mTERF by treatment with the mTERF-specific siRNA, which behaved indistinguishably from mock-transfected cells (Fig. 2B). The overall kinetics of recovery of mitochondrial transcripts compared with cytosolic 5S rRNA was also similar, comparing cells over-expressing mTERF with control cells (Additional File 1, Fig. S2B), and comparing cells knocked down by mTERF-specific siRNA with mock-transfected cells (Additional File 1, Fig. S2C).

### Manipulation of mTERF expression influences both sense- and antisense-strand transcription

**Figure 3:**
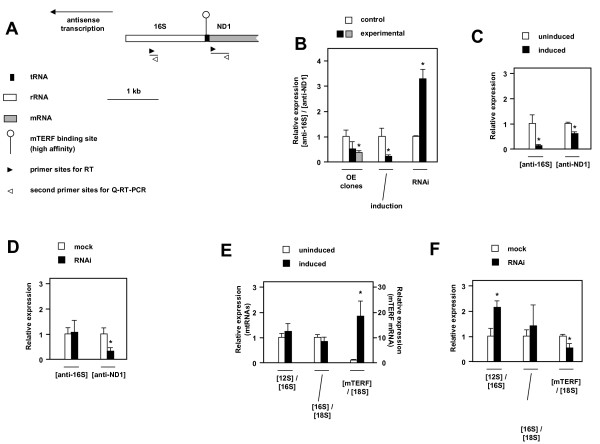
**Manipulation of mTERF expression affects relative levels of antisense transcripts of the 16S rRNA and ND1 genes**. (A) Schematic diagram of 16S rDNA-ND1 region of human mtDNA. For full details of primer sequences and location, see Additional File 1, Table S1. (B) Relative steady-state levels of anti-16S and anti-ND1 transcripts, determined by Q-RT-PCR using proximity probe hybridization (probe sets R1, N1 and C1 for normalization), after various manipulations of mTERF expression, i.e. two mTERF overexpressor clones (OE) compared with vector-transfected cells, doxycyclin-induced *versus *uninduced Flp-In™ T-Rex™-293 cells transfected with mTERF expression construct, and HEK293T cells transfected with mTERF-targeted shRNA *versus *mock-transfected cells. In each case, data were normalized to the corresponding control cells. * denotes statistically significant differences from control cells (*t *test, *p *< 0.02). (C, D) Relative changes in anti-16S and anti-ND1 transcripts, based on replotting of data from the experiment of panel B for each transcript individually, (C) following induced expression of mTERF in Flp-In™ T-Rex™-293 cells and (D) in HEK293T cells transfected with mTERF-targeted shRNA *versus *mock-transfected cells. Data were normalized to values for corresponding untreated control cells, using 18S as internal normalization standard. * denotes statistically significant differences from corresponding control cells (*t *test, p < 0.01). (E, F) Relative steady-state levels of 12S, 16S and 18S sense-strand transcripts, as determined by Q-RT-PCR using of proximity probe hybridization (primer sets T1, R2, C1 respectively, as described in Additional File 1, Table S1), and of mTERF mRNA relative to 18S rRNA (probes sets M1 and C1, see Additional File 1, Table S1), (E) following induced expression of mTERF in Flp-In™ T-Rex™-293 cells and (F) in HEK293T cells transfected with mTERF-targeted shRNA versus mock-transfected cells. * denotes statistically significant differences (*t* test, *p* < 0.02). See also Additional File 1, Fig. S3.

Since the effects of manipulating mTERF expression on the levels of mature 16S rRNA and ND1 mRNA did not reveal any significant changes, we used strand-specific quantitative RT-PCR to analyse effects on the levels of both sense-strand and antisense-strand transcripts derived from specific regions of these genes either side of the high-affinity mTERF binding site. We analyzed the relative levels of antisense transcripts from portions of the ND1 and 16S genes in three contexts in which mTERF expression was manipulated (Fig. 3B). In cell clones stably overexpressing mTERF the relative level of anti-16S to anti-ND1 RNA was decreased compared to control cells transfected with the empty vector, although this difference was only statistically significant in one of the two clones studied. Induction of mTERF expression in Flp-In™ T-Rex™-293 cells stably transfected with the mTERF construct also resulted in a substantial and statistically robust decrease in the anti-16S:anti-ND1 ratio, whereas transfection of HEK293T cells with an shRNA targeted on mTERF resulted in the opposite effect, i.e., a significant large increase in the relative amount of anti-16S RNA. Notably, the pattern of changes in each RNA differed in the two cases (over-expression and knockdown), when comparing its level in treated versus untreated cells (Fig. 3C, D). Induced overexpression, which resulted in a 20-fold increase in mTERF mRNA (Fig. 3E), produced a severe decrease in antisense transcripts from the 16S gene, but also a small decrease in the level of anti-ND1 (Fig. 3C). mTERF knockdown (by a factor of 2 at the RNA level: Fig. 3F) produced no significant effect on antisense transcripts of 16S, but a sharp drop in the level of anti-ND1 (Fig. 3D).

We validated the main findings using a second primer set (Additional File 1, Fig. S3B), which was also used to test effects on the relative amounts of sense-strand transcripts from the 16S and ND1 genes, which were found to be unaffected by these manipulations, as expected from the analysis of mature transcripts by Northern blots (Fig. 1). In addition, we analysed effects on sense-strand transcript from the 12S rRNA gene, and determined the levels of sense-strand transcripts of both mitochondrial rRNAs relative to cytosolic 18S rRNA (Fig. 3E, F). Under conditions of induced over-expression of mTERF, sense-strand transcripts of 12S rRNA and of 16S rRNA were unchanged relative to each other and to cytosolic 18S rRNA (Fig. 3E). However, we did detect a significant *increase *in sense-strand 12S rRNA transcripts in cells knocked down for mTERF (Fig. 3F).

## Discussion

### mTERF and heavy-strand promoter modulation

In this study we investigated the effects of manipulating the expression level of mTERF on the relative levels of different mitochondrial transcripts. Under all conditions tested we failed to detect any significant effects on the relative levels of mature 16S rRNA and ND1 mRNA (Fig. 1C, 1D, Additional File 1, S3C). Over-expression of a tagged mTERF variant, which resulted in the greatest increase in DNA-binding activity that we were able to generate (Fig. 5C of [16]), produced no significant change in the relative levels of the mitochondrial tRNAs tested, with only a minor decrease in their overall abundance (Fig. 1E). Induced 20-fold over-expression of natural mTERF in a controlled nuclear background also did not alter the ratio of mature 16S rRNA to ND1 mRNA, nor were the levels of 16S or ND1 transcripts affected relative to transcripts of cytosolic 18S rRNA or 12S rRNA (Fig. 1C, 3E). Knockdown of mTERF resulted in a very modest decrease in the level of tRNA^Phe ^relative to tRNA^Leu(UUR)^. However, this was not sufficient to generate any significant change in the kinetics of recovery of mitochondrial tRNA levels following EtBr-induced depletion.

We did, however, obtain two piece of evidence that mTERF knockdown is not inert as regards transcription of the mitochondrial heavy strand. Firstly, we observed, by Northern blots, a small increase in the amount of mitochondrial tRNAs belonging to each of the heavy-strand transcription units, relative to cytosolic 5S rRNA (Fig. 1E) in normal cells after mTERF knockdown. Secondly, the level of sense-strand 12S rRNA gene transcripts analysed by quantitative RT-PCR was significantly increased relative to sense-strand 16S rRNA or cytosolic 18S rRNA gene transcripts (Fig. 3F), in normal cells knocked down for mTERF. However, the levels of sense-strand 16S and ND1 transcripts relative to each other or to 18S were not significantly affected (Fig. 3F). This suggests the existence of a compensatory mechanism, whereby decreased mTERF levels, which might otherwise impair 16S rRNA biogenesis, generate a signal for globally increased mitochondrial transcription (or decreased turnover) to overcome any such defect. It may also be noted that the effects of knockdown may be underestimated due to the rather limited decrease in mTERF mRNA level that we were able to achieve in these experiments. A 50% decrease is not untypical in cultured mammalian cells in cases where knockdown of a given gene may provoke a growth defect, even just a transient one, compared with untransfected cells in the culture. Thus, the effects we observed may likely represent a combination of normal expression in almost half the cells, plus greatly reduced expression in the remaining cells.

Nevertheless, our findings imply that the expression level of mTERF does not determine, in a simple manner, the relative steady-state levels of transcripts belonging to the two transcription units of the heavy-stand. Although mTERF was previously shown to stimulate transcription *in vitro *from PH1 in a comparatively crude system [12,18], it may be noted that no such effect was seen when purified, recombinant proteins were used [15], or even in crude extracts using DNA-affinity purified mTERF [18].

Our results indicate that even if mTERF levels do influence transcriptional readthrough, a compensatory response nevertheless adjusts the relative output of different transcripts belonging to the two heavy-strand transcription units. This may involve the modulation of transcriptional initiation, post-transcriptional processing or RNA turnover. Our findings are consistent with previous reports of the action of thyroid hormone [36] or variation in ATP supply [37], both of which can influence the relative rates of transcriptional of initiation at PH1 and PH2 without any effect on that at the high-affinity mTERF binding site. It is also possible that mTERF might have a different physiological function, and that its effects on transcription are accommodated by modulating other components of the mitochondrial RNA synthesis machinery.

### Is mTERF activity in HEK293 cells physiological?

All of the current study was conducted in one cell-line and its derivatives which, as a cancer cell-line, may not behave in a physiologically normal manner. We considered the hypothesis that mTERF levels may, in other cell-types, have a more profound effect on mitochondrial transcription but that, in HEK293 cells, mTERF could be present in such excess that neither over-expression nor any amount of knockdown achievable by RNAi technology influences its functional level. However, from available gene expression data (biogps.gnf.org) the range of expression of mTERF in different cell-types *in vivo*, plus primary tumours and cell-lines including HEK293 and its derivatives, is only of the order of 2-5 fold. Furthermore, in HEK293T cells mTERF is expressed at very close to the median level for all cells investigated. Therefore, the range of expression achieved in the present study (~40-fold at the RNA level, Fig. 3E, F) far exceeds that known to be experienced *in vivo*.

Another possibility, given the wealth of previous data indicating possible post-translational regulation of the transcriptional activity of mTERF, is that mTERF is constitutively inactivated in HEK293T cells, regardless of its expression level. Although we analysed DNA-binding activity as well as RNA levels, some mTERF preparations that are competent for DNA binding are nevertheless unable to influence transcription *in vitro *[11,24]. This is unlikely, however, since the patterns of mitochondrial transcripts in HEK cells, and their responses to other manipulations, such as increases in the level of TFAM [38], are similar to other cultured cells and *in vivo *tissues.

Thiamphenicol treatment, which alters the representation of PH1- and PH2-derived transcripts in a manner similar to thyroid hormone treatment, is able to modify the EMSA signal at the high-affinity mTERF binding site, whilst leaving the actual levels of mTERF polypeptide unaffected [39]. This may indicate that a post-translational modification of mTERF could modulate both its DNA-binding and its transcriptional properties *in vivo*, but is equally consistent with the notion that another factor, capable of binding in this region, is involved.

Final resolution of these issues will require the creation of an *in vivo *model in which mTERF levels can be manipulated over at least as great a range in a tissue-selective manner. The possibility of redundancy between mTERF and other members of the mTERF family in regulating read-through transcription at the 16S/tRNA^Leu(UUR) ^gene boundary needs also to be considered.

### Modulation of antisense-strand transcripts

We found that alterations in mTERF expression produced systematic changes in the extent of read-through transcription in the antisense direction, as inferred from the relative levels of anti-16S to anti-ND1 transcripts. Increased levels of mTERF, resulting from stable over-expression or from induction of Flp-In™ T-Rex™-293 cells transfected with an mTERF expression construct, shifted the balance of antisense transcripts in the anti-ND1 direction, whereas mTERF knockdown had the opposite effect, shifting the balance in favour of anti-16S. These findings are consistent with the notion that mTERF, bound to its high affinity binding site in the tRNA^Leu(UUR) ^gene, promotes termination of antisense transcription initiated at PL, which has traversed most of circular genome. Increased termination at this site should deplete the representation of anti-16S, whereas decreased termination should increase the amount of anti-16S, consistent with our observations. However, the effects seen are more complex than implied by this simple model. Specifically, the shift towards anti-ND1 under conditions of over-expression consists of a rather drastic decrease in the amount of stable anti-16S, combined with a much smaller decrease in the amount of anti-ND1 (Fig. 3E). Since there are additional, weaker binding sites for mTERF in the IQM tRNA cluster and ND1 coding sequence [16], our finding supports the idea that a high level of mTERF leads to increased occupancy also of these weaker affinity binding sites, restraining readthrough into anti-ND1 as well as the more dramatic effect on readthrough into anti-16S further downstream. On the other hand, mTERF knockdown resulted in a clear decrease in the level of anti-NDI1 but only a small change in anti-16S (Fig. 3F). These findings imply that maintenance of the physiological level of mTERF is important for the formation of stable antisense transcripts of ND1, by preventing readthrough into the rDNA. If this interpretation is correct, one *in vivo *role of mTERF is thus inferred to be the regulation of antisense transcriptional termination, for an unknown physiological reason.

*In vitro*, mTERF exhibits bidirectional termination activity [15]. If this applies also *in vivo*, it may be that the primacy of post-transcriptional processing, the stabilization of rRNA into ribosomal subunits, and compensatory effects on transcriptional initiation or RNA stability, mask or complicate the effects on sense-strand transcripts. Conversely, antisense transcripts, which are destined only for turnover (or for some unknown physiological function) would appear to be regulated more straightforwardly by mTERF.

A somewhat different interpretation arises from the recent, and thus far unexplained reports of hairpin-loop transcripts deriving from the 16S rRNA gene, whose levels appear to reflect the proliferation status and tumorigenicity of cells [40,41]. It is not yet known how these transcripts arise. Possibilities are that they are created post-transcriptionally by *trans*-splicing or RNA ligation, or else that they arise by template strand-switching during transcription. Our antisense results could thus imply that mTERF influences the rate of their production in ways related to or even independent of its binding to mitochondrial DNA.

### Physiological function(s) of mTERF

Given that the effects of mTERF manipulation on the levels of mature mitochondrial transcripts *in vivo *appear to be negated or modified by compensatory mechanisms, it may be that the principal physiological function of this evolutionarily conserved protein is something other than transcriptional regulation as such. In our previous study [16] we speculated that mTERF might play some role in regulating collisions between oppositely moving transcription and replication machineries, facilitating their orderly passage, whilst minimizing the risk of stalled replication giving rise to recombinogenic 3' ends. A requirement for such activity is well established in both prokaryotic and eukaryotic DNA replication [42,43], and other members of the mTERF family have been inferred to play a role in the completion of DNA replication in human cells [44]. The presence of a transcriptional terminator at a replication pause site moreover provides a potential primer of lagging-strand synthesis commencing immediately from the pausing site, ensuring that no region remains single-stranded and hence susceptible to DNA damage during pausing. The RITOLS model of mtDNA replication [45] postulates that the entire lagging strand is laid down initially as RNA, which might be facilitated by such a mechanism. However, the lagging strand for mtDNA replication is the same strand as the rRNA. Therefore, if bound mTERF were to deliver the 3' end of a paused transcript to an arriving replication complex, this would be as a result of its activity in the sense direction. The role of attenuation on the antisense strand is less clear, although this might provide a primer required for re-initiation of the replication machinery at a stalled replication fork, especially since the former leading strand 3' end may be unavailable, e.g. due to fork regression. A role for DnaG primase in replication restart at stalled, gapped forks has been identified in *E. coli *[46], serving as a precedent for primer-dependent restart. Codirectional collisions between the transcription and replication machineries in *E*. coli also generate leading-strand gaps, with the nascent RNA being recruited as a new primer by the replisome [47].

Another possibility which should be seriously considered is that mTERF's effects on nucleic acid metabolism are incidental to its real biological function inside mitochondria, which may be something completely different. However, arguing against this is the fact that other members of the mTERF family also affect mitochondrial transcript levels, including a recently reported case of the SOLDAT10 protein in *Arabidopsis *chloroplasts, a mutation in which appears to activate retrograde signaling by decreasing plastid rRNA synthesis [48]. MOC1, an mTERF family homologue in *Chlamydomonas*, is required for maintaining mitochondrial RNA levels after exposure to light, although its mechanism of action is unknown and the broader phenotype of the mutant suggests that the effect might be indirect [49].

## Conclusions

In summary, our findings support a role for mTERF in influencing mitochondrial transcription *in vivo*, even though it does not appear to set the levels of mature mitochondrial transcripts encoded by the PH1 and PH2 heavy-strand transcription units in a simple manner. It appears to modulate the levels of antisense transcripts, by implication regulating the extent of readthrough by the transcriptional machinery of its high-affinity binding site in the tRNA^Leu(UUR) ^gene, as well as other, weaker mTERF binding sites in the vicinity. Further experiments will now be required to resolve the functional significance of this regulation, and its possible relevance to DNA replication and other processes.

## Methods

### Cell-lines and cell culture

HEK293T cells and derivatives were cultured in Dulbecco's modified Eagle's medium (DMEM, Sigma) as previously [16]. HEK293T-derived cell-clones over-expressing natural mTERF were created by recloning the mTERF coding sequence, including its natural stop codon, into the expression vector pcDNA3.1/hygro(-) (Invitrogen) as a *Bam*HI/HindIII fragment. Aliquots of the sequence-verified plasmid DNA (1 μg) were transfected into HEK293T cells using Lipofectamine™ (Invitrogen) diluted in 1 ml of Opti-MEM^® ^(Invitrogen) according to the manufacturer's protocol. Twenty four hours later cells were either harvested (for transient transfection) or placed under hygromycin selection (Calbiochem, 200 μg/ml). Hygromycin-resistant colonies were grown up and tested for expression of the mTERF transgene by reverse transcriptase (RT)-PCR and by electrophoretic mobility shift assay (EMSA) as described in Additional File 1. Flp-In™ T-Rex™ 293 cells transfected with expression constructs for natural mTERF and for epitope-tagged mTERF-MycHis, as well as their induction by doxycycline, were as described previously [16]. mTERF-specific RNA interference was induced by siRNA for 48 h as described previously [16] or by transfection (using Lipofectamine™ 2000, Invitrogen, manufacturer's protocol) with a customized shRNA construct (10 μg) targeting the following sequence within mTERF mRNA (5' to 3'): GCUGUAACUUGAGUACUUU, Open Biosystems Expression Arrest™ pSM2 Retroviral shRNAmir Library, Oligo ID V2HS_95064 (Thermo Fisher Scientific, Huntsville, AL, USA). shRNA-transfected cells were harvested 48 h after transfection.

### Depletion of mitochondrial RNA

Cells were passaged one day before adding ethidium bromide (EtBr) so that the 60 × 15 mm plates were approximately 50% confluent on the day of experiment. EtBr was added to the medium to 250 ng/ml and the cells were incubated for 48 h, after which the plates were approximately 90% confluent. Cells were then passaged at different densities so that each re-seeded plate would reach approximately 70-80% confluence when harvested for RNA extraction. RNA samples were collected before EtBr treatment (day -2), on the day when drug was washed away (day 0) and 24, 48, 72, 96 and 120 h after removing EtBr (days 1-5). To ensure complete removal of EtBr the medium was changed 3 h and 6 h after passaging the cells, and then again every day. Where depletion was carried out in combination with mTERF-directed RNA interference, siRNA transfection was carried out prior to the addition of EtBr (day -2) and was repeated 2 d after removal of the drug (day 2).

### RNA extraction, electrophoresis and Northern blotting

Total RNA was extracted from cells using TRIzol^® ^Reagent (Invitrogen) according to the manufacturer's instructions. Any traces of DNA were removed by treatment with RNase-free DNase I (GE Healthcare, manufacturer's recommended conditions), followed by standard acid phenol/chloroform extraction and isopropanol precipitation. For Northern blotting to tRNA probes RNA samples were electrophoresed at 4°C overnight at 100 V in neutral 12% acrylamide/7 M urea gels in TBE buffer, electroblotted onto Zeta-Probe GT membrane (Bio-Rad) at 4°C, u.v.-crosslinked and processed as described previously [50]. Oligonucleotide probes for mitochondrial tRNAs and cytosolic 5S rRNA were radiolabeled using T4 polynucleotide kinase (PNK, MBI Fermentas) according to the manufacturer's protocol and [γ-^32^P] ATP (Amersham Pharmacia Biotech, 3000 Ci/mmol) and purified using mini Quick Spin Columns (Roche). The probe oligonucleotide sequences were as follows (all 5' to 3'): 5S - GGGTGGTATGGCCGTAGAC, tRNA^Leu(UUR) ^- GTTTTATGCGATTACCGGGC and tRNA^Phe ^- CTAAACATTTTCAGTGTATTGC. Hybridization, washing, autoradiography and phosphorimaging (Phosphorimager SI, Molecular Dynamics) were as described previously [51]. For re-probing, the membranes were stripped by boiling in 0.5% SDS solution for 3 min and cooled to room temperature. For Northern blotting to 16S rRNA or ND1 probes, RNA samples were fractionated on formaldehyde agarose gels and processed for blotting and hybridization as described previously [51], using probes labelled by random-priming [50]. The template used for synthesis of the ND1 probe was as described previously [50]; that for 16S rRNA was the shorter *Apa*I digestion product (230 bp) from the same fragment.

### Quantitative RT-PCR

Quantitative RT-PCR was used to estimate the relative amounts of 12S and16S rRNA, ND1 mRNA, cytosolic 18S rRNA and mTERF mRNA. For cDNA synthesis, 5 μg of RNA was reversed transcribed using 40 units of M-MuLV reverse transcriptase (Fermentas), primed by 0.2 μg random hexamers (Pharmacia) in a 20 μl reaction according to manufacturer's instructions. Three dilutions of each cDNA sample (1:10, 1:20 and 1:50) were analysed, and each reaction was performed in three technical replicates. PCR reactions were performed in a LightCycler™ apparatus using LightCycler FastStart DNA Master SYBR Green I kit (Roche) according to the manufacturer's instructions, with the following primer pairs (all 5' to 3') and annealing temperatures: for 18S rRNA, 18Sfor3 - GACGATCAGATACCGTCGTA and 18Srev3 -TGAGGTTTCCCGTGTTGAGT, 52°C; for 16S rRNA, 16Sfor1 - GGTAGAGGCGACAAACCTACCG and 16Srev1 - TTTAGGCCTACTATGGGTGT, 50°C; for ND1 mRNA, ND1for1 - GGCCAACCTCCTACTCC and ND1rev1 - GATGGTAGATGTGGCGGGTT, 50°C. cDNA synthesized from 5 μg of RNA pooled from different cell-lines was used to prepare the standard curve, based on a five-fold dilution series. The homogeneity of all products was checked after each run by melting curve analysis. For strand-specific analysis to distinguish antisense from sense transcripts, 20 pmol of specific primer (TIB MOLBIOL, Berlin, Germany, see Additional File 1, Table S1) were used in the RT step. The PCR step used custom-designed sets of primers and proximity-hybridization probes (TIB MOLBIOL, Berlin, Germany, see Additional File 1, Table S1), with LightCycler(R) FastStart DNA Master HybProbe kit (Roche), according to manufacturer's instructions, and annealing temperatures listed in Additional File 1, Table S1 for each primer pair. The homogeneity of the products was checked after each run by melting curve analysis, according to the annealing temperatures of the hybridization probes as listed in Additional File 1, Table S1. Three dilutions (1:10, 1:20 and 1:50) were analysed from each cDNA. The level of mTERF mRNA relative to 18S rRNA was measured similarly, using hybridization probe sets M1 and C1 (see Additional File 1, Table S1), except that cDNA primed with random hexamers was used as template.

## Authors' contributions

AKH performed the experimental work, assisted by MKK for Q-RT-PCR, analyzed the data and co-drafted sections of the manuscript (Results, Materials and Methods, Figure Legends). SKM co-designed the project and co-supervised its initial stages. HTJ co-designed and supervised the project, compiled the figures and drafted the manuscript. All authors saw and approved the final version of the manuscript.

## Supplementary Material

Additional file 1 **Supplementary text, Table (S1) and Figures (S1, S2, S3)**. All supplementary data is supplied as a single PDF file containing the following items: Supplementary Methods, Legends to Supplementary Figures, Supplementary Table (Table S1), Supplementary Figures S1, S2 and S3. Click here for file
