# Supplementary material for: Effects on mitochondrial transcription of manipulating mTERF protein levels in cultured human HEK293 cells
Source: BMC Mol Biol. 2010 Sep 16;11:72. doi: 10.1186/1471-2199-11-72 (PMC2955023; doi:10.1186/1471-2199-11-72)
Supplement: Additional file 1 — Supplementary text, Table (S1) and Figures (S1, S2, S3). All supplementary data is supplied as a single PDF file containing the following items: Supplementary Methods, Legends to Supplementary Figures, Supplementary Table (Table S1), Supplementary Figures S1, S2 and S3. [file 1471-2199-11-72-S1.PDF]

Effects on mitochondrial transcription of manipulating mTERF protein levels in cultured human HEK293 cells

Anne K. Hyvärinen, Mona K. Kumanto, Sanna K. Marjavaara, & Howard T. Jacobs

ADDITIONAL FILE 1

SUPPLEMENTARY METHODS

Primer and probe sets for Q-RT-PCR using proximity-hybridization probes

See this file, Table S1.

Verification of mTERF transgene expression by RT-PCR

Ten μg of total RNA was used for cDNA synthesis using random hexamers (Pharmacia) and M-MLV reverse transcriptase (Invitrogen) in a 20 μl reaction (manufacturer's recommended conditions). For PCR 2 μl of the RT reaction mix was used in a 25 μl reaction containing 0.4 mM dNTPs (Fermentas), 0.4 μM of both primers and 2 u of Dynazyme DNA polymerase (Finnzymes). Transgene-specific primers were (all 5´ to 3´): (BGH – TAGAAGGCACAGTCGAGGC and mTERF465F – CGAGCAATAACACGTACTCC; 18S specific primers were: 18S-F – TACCTGGTTGATCCTGCCAG and 18S-R – TCGGGAGTGGGTAATTTGC. To exclude possible contaminating DNA, PCR with 18S primers was routinely carried out on each RNA sample, alongside a DNA positive control.

Verification of mTERF transgene expression by EMSA

EMSA was carried out as described previously [16], using probe 'Leu-short' for the mTERF high-affinity binding site, 5 µg of protein in mitochondrial lysate and 5 µg of non-specific competitor poly(dI-dC)–(dI-dC) (Amersham Pharmacia Biotech).

Western blotting to confirm induction of mTERF-MycHis

Western blotting to detect the mTERF-MycHis fusion protein in Flp-In^{TM} T-REx^{TM}-293 cells transfected with the mTERF-MycHis construct was performed as previously [16].

Image processing

Western blot and gel images are cropped to show relevant bands. In some cases brightness and contrast were adjusted to make the images optimally visible, but no gamma correction was performed and no other manipulations were done. Non-adjacent tracks of the same gel are shown separately, alongside each other.

SUPPLEMENTARY FIGURE LEGENDS

Figure S1

Manipulation of mTERF expression and effects on steady-state levels of mature mitochondrial RNAs

(A) Verification of expression of mTERF transgene in transfected cell clones, by RT-PCR, using primers specific for the transgene (mTERF 465F and BGH), and for 18S rRNA (18S-F and 18S-R) as loading control, as described in Supplementary Materials and Methods. RNA was extracted from clones, numbered as shown, of hygromycin-resistant cells transfected with the natural mTERF expression construct described previously [16]. – denotes water control, H untransfected HEK293T cells and M the marker ladder. Clone 23 is included here as an example of a clone negative for expression, which was not selected for use in the experiment shown in Fig. 1B. Panels shown alongside each other are aligned, non-adjacent tracks from the same gel. (B) Overexpression of mTERF at the protein level was verified by EMSA using a probe for the high-affinity mTERF binding site, together with mitochondrial protein extracts from clones, numbered as shown, of hygromycin-resistant cells transfected with the mTERF expression construct, plus v – empty vector-transfected cell clone and tr – cells transiently transfected with the mTERF expression construct. – denotes buffer-only control. Clone 7 is included here as an example of a clone negative for expression at the protein level, which was not selected for use in the experiment shown in Fig. 1B. The unbound and bound probe migrated as indicated. The specificity of binding was verified as previously, using cold competitor. Panels shown alongside each other are aligned, non-adjacent tracks from the same (two) gels. (C) Northern blots of RNA samples as indicated, probed for mitochondrial $tRNA^{Phe}$, $tRNA^{Leu(UUR)}$ and cytosolic 5S rRNA as shown. Data from the blots is compiled as Fig. 1E, based on phosphorimaging. The three equivalent samples loaded alongside each are independent RNA preparations from biological replicates.

Figure S2

Effects of mTERF overexpression on mitochondrial transcript levels during EtBr-induced depletion and recovery

Representative series of Northern blots probed successively for mitochondrial $tRNA^{Phe}$ and $tRNA^{Leu(UUR)}$ and for 5S rRNA as indicated. Similar sets of blots were used to compile phosphorimager data for the graphs shown in (B) and (C) and in Fig. 2. RNA was from mTERF overexpresor (OE) clone 3, as characterized in Fig. S1, and the same empty vector-transfected clone as used in the experiment of Fig. S1B. Day -2 indicates sample taken immediately before addition of EtBr to the culture to induce depletion of mitochondrial RNA. Day 0 indicates sample taken 48 h later, immediately after EtBr was washed out from the culture. Days 1-5 indicate the period of subsequent recovery. (B), (C) Relative expression of mitochondrial transcripts in cells overexpressing mTERF, based on phosphorimaging of Northern blots probed successively for mitochondrial $tRNA^{Phe}$, $tRNA^{Leu(UUR)}$ and 5S rRNA. Data (means $\pm$ SD) are ratios of $tRNA^{Phe}$ to 5S rRNA (F/5S) normalized to the ratio at the start of the experiment (time-point -2 d). (B) Cells stably transfected with empty-vector or mTERF overexpression (OE) construct (clone 3, as shown in Fig. S1), sample blots shown in Fig. S2A. (B) Cells treated with mTERF-specific siRNA (or mock-transfected) prior to the addition of EtBr (day -2) and again 2 days after removal of EtBr (day 2). Days 1-5 indicate the period of subsequent recovery. For equivalent data on ratio of $tRNA^{Phe}$ to $tRNA^{Leu(UUR)}$ from the same experiment see Fig. 2. Note that this experiment only measures *changes* in the tRNA ratio during the experiment, but does not allow to extrapolate an absolute ratio of the two tRNAs, since the hybridization efficiency of the two probes may differ. Moreover, to control rigorously for loading differences we used the same blot in each case for reprobing after stripping. Impressionistically, we found nothing to contradict the findings of King and Attardi (*J. Biol. Chem*. 268:10228;1993) that $tRNA^{Phe}$ is expressed normally at a higher level than $tRNA^{Leu(UUR)}$.

Figure S3

Manipulation of mTERF expression affects the relative levels of antisense but not sense transcripts of the 16S rRNA and ND1 genes

(A) Schematic diagram of the 16S rDNA and ND1 region of human mtDNA. For full details of Q-RT-PCR primer sequences and location, see Table S1. The same primer sets were used to assay levels of sense and antisense transcripts, except that reverse transcription step was carried out with the relevant strand-specific primer in each case. (B) Relative steady-state levels of anti-16S and anti-ND1 transcripts, as determined by Q-RT-PCR (primer sets R2 and N2), using hybridization of proximity probes, under various manipulations of the level of mTERF, i.e. the same mTERF overexpressor clone (OE) that exhibited a statistically significant decrease in anti-16S:anti-ND1 ratio using primer sets R1 and N1 (Fig. 3B), compared with cells transfected with empty vector, and HEK293T cells transfected with mTERF-targeted shRNA *versus* mock-transfected cells. In each case, data were normalized to the corresponding control cells. * denotes statistically significant differences from control cells (*t* test, $p < 0.02$). The alterations in the anti-16S:anti-ND1 ratio were qualitatively similar to those obtained in similar experiments using primer sets R1 and N1 (Fig. 3B), although quantitatively slightly lower. (C) As an additional control we validated the earlier findings regarding sense transcripts, using Q-RT-PCR with primer sets R2 and N2 combined with hybridization of proximity probes, under the various manipulations of the level of TERF used in the corresponding experiment on antisense transcripts (Fig. 3B). None of the comparisons showed any significant difference (*t* test, $p > 0.05$). To validate the primer sets and RNA preparations used in the Q-RT-PCR experiments of Fig. 3B and Fig. S3B, C, we conducted a series of controls illustrated by the gels shown in (D). Firstly, the 16S and ND1 primer sets used for reverse transcription and PCR were tested alongside total cell DNA to verify that they gave rise only to a single PCR product of the correct size when cDNA primed with either primer of the set was amplified subsequently amplified with both (panels *i* and *ii*). This was

also checked by melting-curve analysis after PCR and proximity-probe hybridization. Secondly, the purity of each RNA preparation was checked by standard PCR, using the primer pairs employed, plus a primer pair for 18S rDNA, as shown here (panel *iii*), and thus shown to be free of DNA contamination. Thirdly, we excluded that the failure to detect such contaminating DNA was due to inhibition by RNA in the sample, by repeating the analysis after the RNA had been treated with boiled RNase A (panel *iv*). The shRNA construct used in the experiments shown in Fig. 3B and Fig. S3B, C differs from the siRNA used in the earlier experiments. Therefore, prior to use in this context, the effects of transfection of the shRNA were validated using doxycyclin-induced Flp-In™ T-Rex™-293 cells transfected with the tagged mTERF-MycHis expression construct [16] and Western blotting. As shown (E), shRNA constructs mTERF.1 and 5 both effected knockdown, and shRNA mTERF.1 (the construct described under Materials and Methods in the main paper) was selected for use in the experiments shown in Fig. 3B and S3B, C. Note that the knockdown experiments in the main paper, shown in Fig. 3, used normal HEK293T cells, not mTERF-MycHis transfected cells. The latter were used only for initial selection of the appropriate shRNA construct.

SUPPLEMENTARY TABLES

Table S1

**Primer/probe set**	**Primer/probe ID, purpose**	**Sequence (5´ to 3´)**	**Annealing Temp (°C)**
R1	Humit16S-fw, primer on antisense template	GGTAGAGGCGACAAACCTACCG	55
	Humit16S-as, primer on sense template	TAGTGGGTGTTGAGCTTGAACG	55
	Humit16S-FL, probe	GGTTCTGTGGGCAAATTTAAAGTTGAACTAAGA-FL	60
	Humit16S-LC, probe	LC640-TCTATCTTGGACAACCAGCTATCACCAGG-P	60
N1	mtND1-se, primer on antisense template	CCAACCTCCTACTCCTCATTGTAC	51
	mtND1-rev, primer on sense template	GATGGTAGATGTGGCGGGTT	51
	mtND1-FL, probe	GGGCCTTTGCGTAGTTGTATATAGCCT-FL	57
	mtND1-LC, probe	LC640-GAATTTTTCGTTCGGTAAGCATTAGGAAT-P	57

R2	16S F, primer on antisense template	AGAGAGTAAAAAATTTAACACCCAT	47
	16S A, primer on sense template	TTCTATAGGGTGATAGATTGGTCC	47
	16S FL, probe	AAGCTCAACACCCACTACCTAAAAAA-FL	55
	16S LC, probe	LC640-CCCAAACATATAACTGAACTCCTCACACC-P	55
N2	ND1_F, primer on antisense template	CCTCATTGTACCCATTCTAATC	45
	ND1_R, primer on sense template	CGTAGTTTGAGTTTGATGCT	45
	ND1_FL, probe	CGCCACATCTACCATCACCCTCTACA-FL	60
	ND1_LC, probe	LC640-CACCGCCCCGACCTTAGCTCT-P	60
T1	12SF101, primer on antisense template	TAGAGGAGCCTGTTCTGTAATCGA	52
	12SB211, primer on sense template	TGCGCTTACTTTGTAGCCTTCAT	52
	12S FL, probe	CGATCAACCTCACCACCTCTTGCTC-FL	60
	12S LC640, probe	LC640-CCTATATACCGCCATCTTCAGCAAACCC-P	60

M1	MTERF S, primer for sense strand	GCAGAGCCTTTCCTTAGGAC	50
	MTERF A, primer for antisense strand	GTCATCCAACATCTTGAACCAA	50
	MTERF FL, probe	AGGTTTCCTGGTGCCATAATGGT-FL	56
	MTERF LC, probe	LC640-AGGTAGTTCAAACCTTTTGAAATGCTTGT-P	56
C1	18S for, primer on antisense template	ACGRACCAGAGCGAAAGCAT	52
	18S rev, primer on sense template	GGACATCTAAGGGCATCACAGAC	52
	18S FL, probe	TCGGAACTACGACGGTATCTGATCGTC-FL	59
	18S LC, probe	LC640-CGAACCTCCGACTTTCGTTCTTGAT-P	59

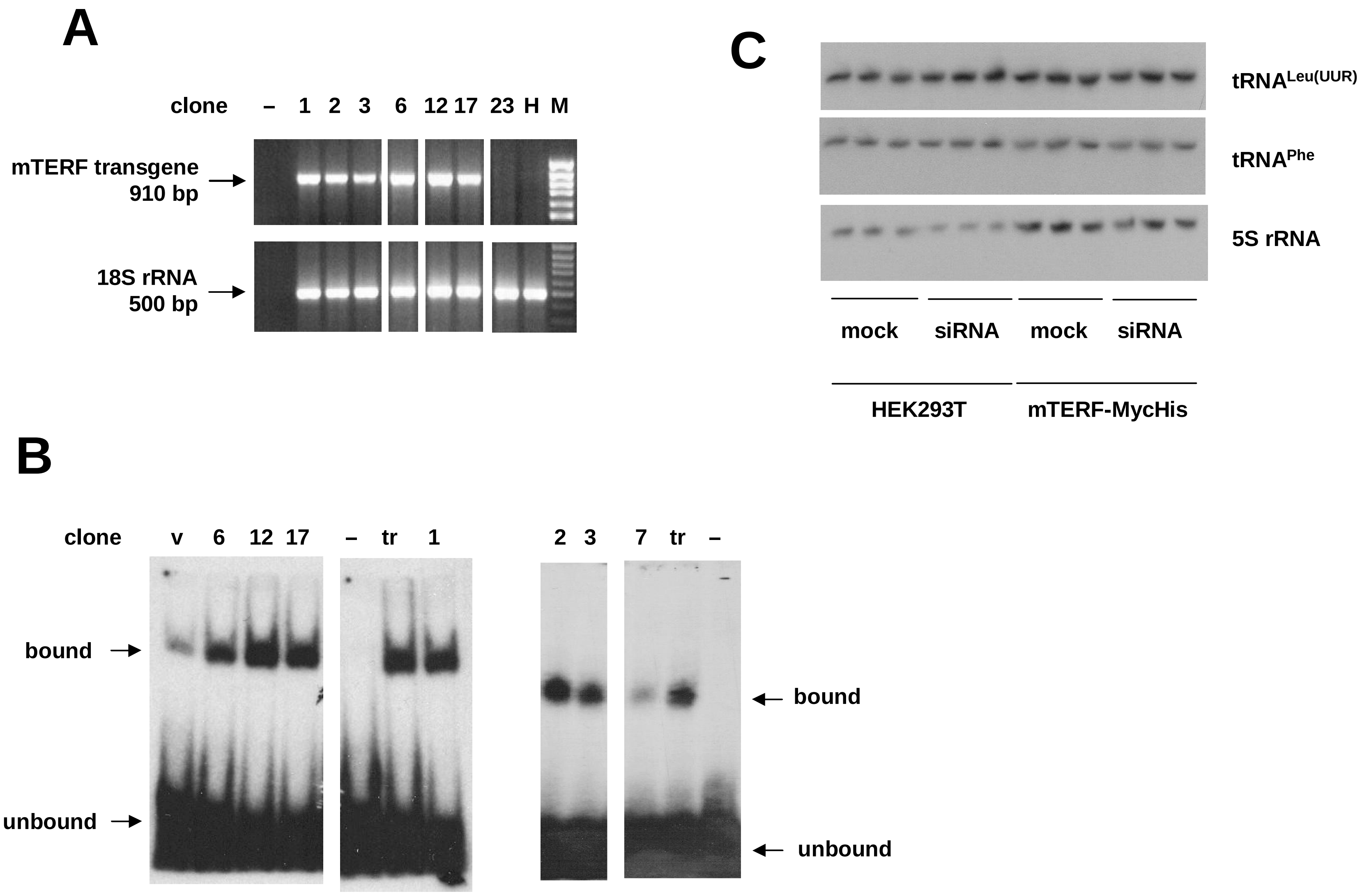

Hyvärinen et al., Figure S1

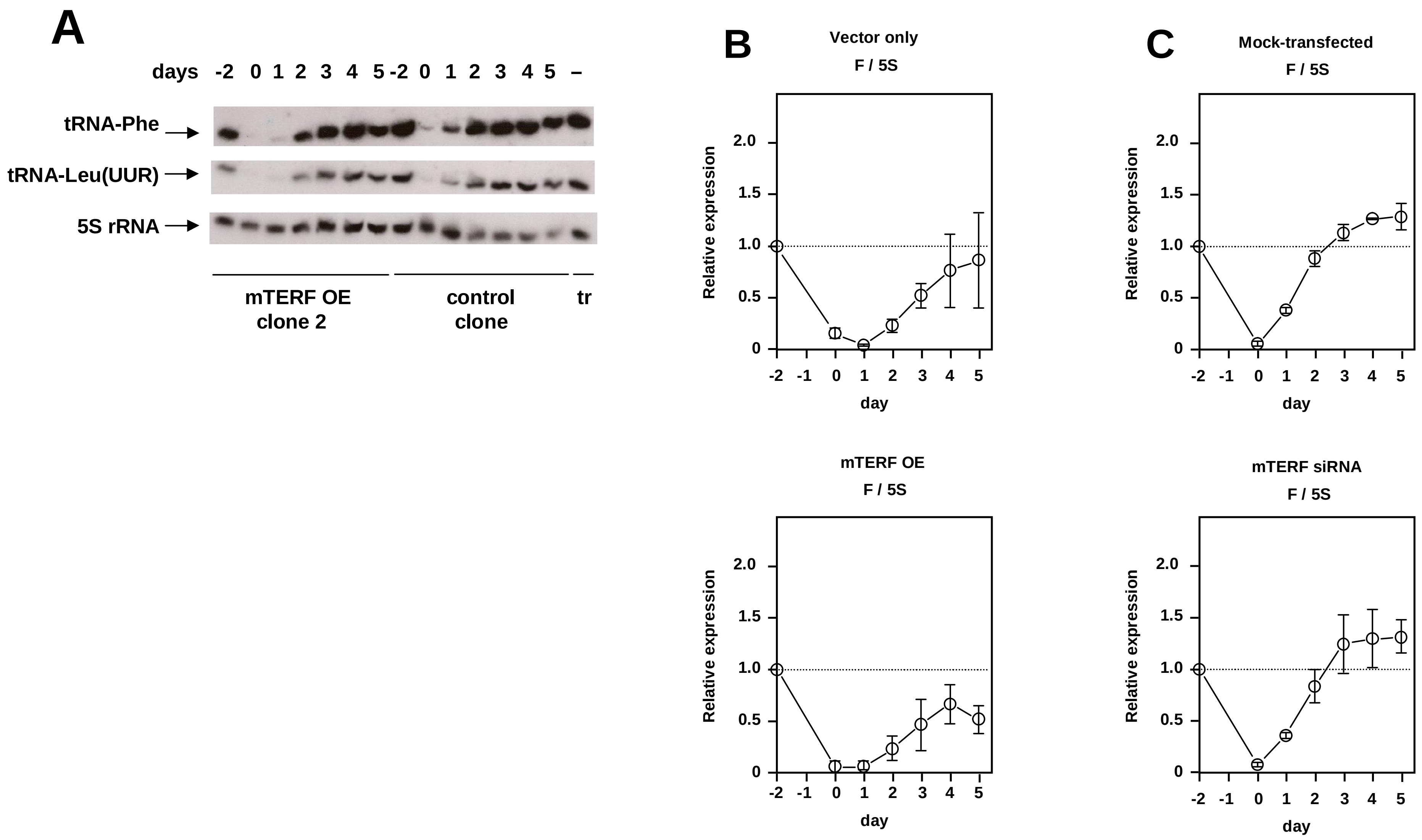

Hyvärinen et al., Figure S2

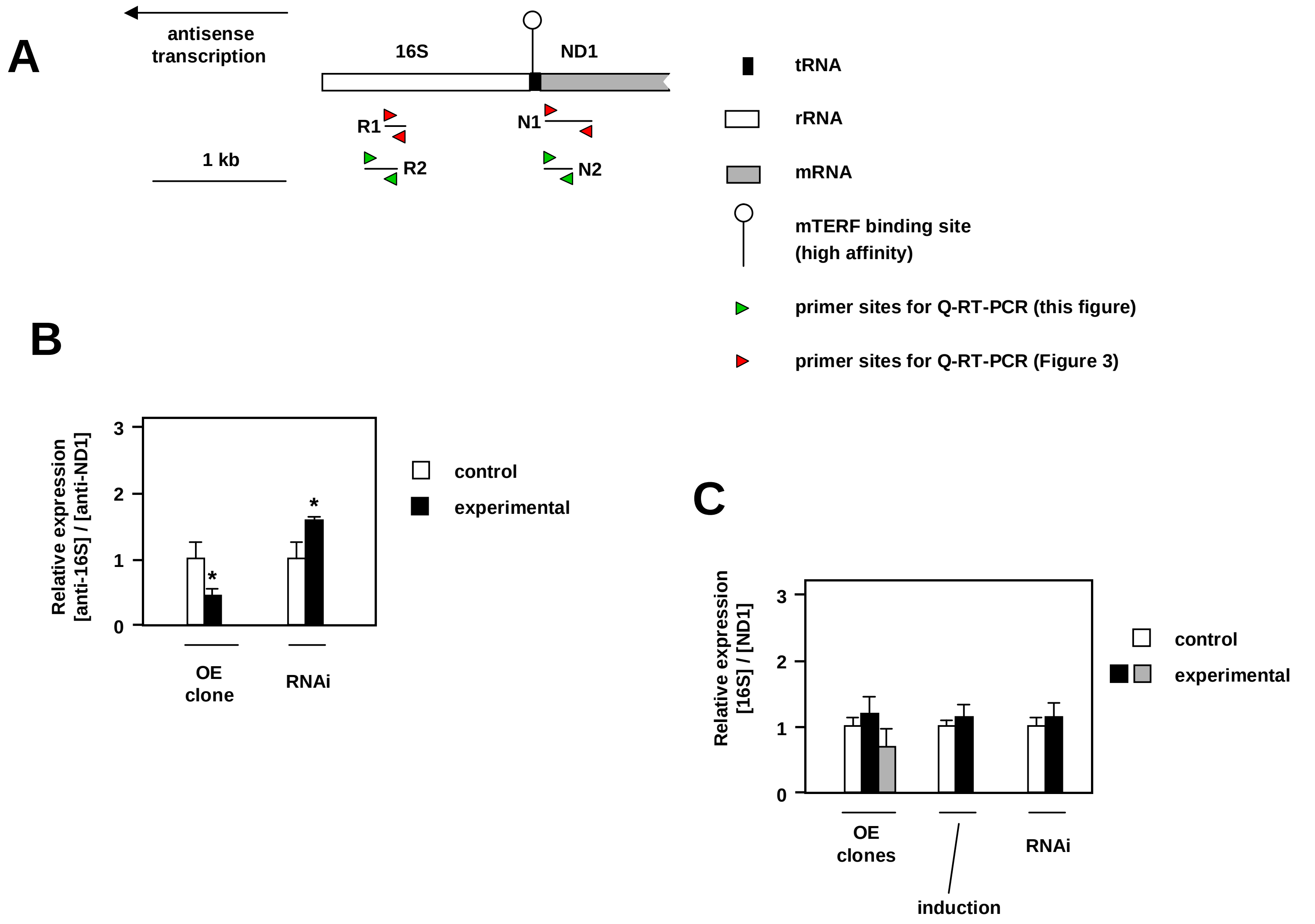

Hyvärinen et al., Figure S3. page 1 of 2

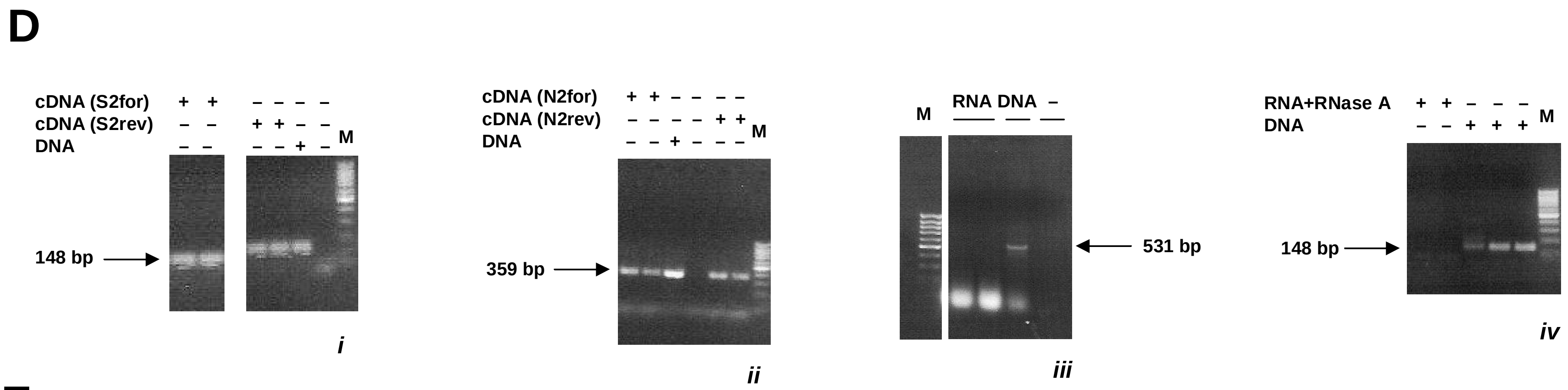
D
cDNA (S2for)
cDNA (S2rev)
DNA
M
148 bp
i
cDNA (N2for)
cDNA (N2rev)
DNA
M
359 bp
ii
M
RNA DNA –
531 bp
iii
RNA+RNase A
DNA
M
148 bp
iv

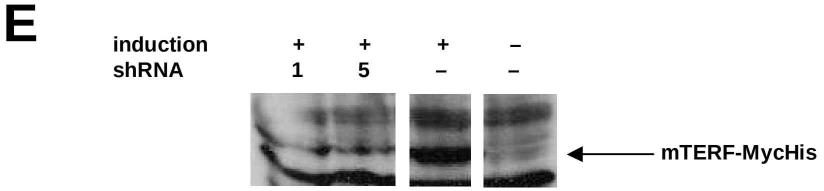
E
induction
shRNA
1
5
mTERF-MycHis
